# Surface engineering of perovskite films for efficient solar cells

**DOI:** 10.1038/s41598-017-14920-w

**Published:** 2017-11-03

**Authors:** Jin-Feng Wang, Lei Zhu, Ben-Guang Zhao, Yu-Long Zhao, Jian Song, Xiu-Quan Gu, Ying-Huai Qiang

**Affiliations:** School of Materials Science and Engineering, China University of Mining and Technology, Xuzhou, 221116 China

## Abstract

It is critical to prepare smooth and dense perovskite films for the fabrication of high efficiency perovskite solar cells. However, solution casting process often results in films with pinhole formation and incomplete surface coverage. Herein, we demonstrate a fast and efficient vacuum deposition method to optimize the surface morphology of solution-based perovskite films. The obtained planar devices exhibit an average power conversion efficiency (PCE) of 13.42% with a standard deviation of ±2.15% and best efficiency of 15.57%. Furthermore, the devices also show excellent stability of over 30 days with a slight degradation <9% when stored under ambient conditions. We also investigated the effect of vacuum deposition thickness on the electron transportation and overall performance of the devices. This work provides a versatile approach to prepare high-quality perovskite films and paves a way for high-performance and stable perovskite photovoltaic devices.

## Introduction

Organometal halide perovskites (*e.g*., CH_3_NH_3_PbI_3_ (MAPbI_3_), CH_3_NH_3_PbCl_3_ (MAPbCl_3_)) have emerged as absorber materials for thin-film photovoltaic applications due to their desirable optoelectronic properties including high absorption coefficient, long exciton diffusion length, long carrier lifetime, high carrier mobility, tunable band gap and apparent tolerance of defects^1–7^. Recently, perovskite solar cells (PSCs) have been reported to achieve a certified efficiency of 22.1%, showing a potential to compete with traditional silicon photovoltaic industry in the near future^8^.

Compared with MAPbI_3_, MAPbI_x_Cl_3−x_ perovskite possess a higher charge carrier mobility and longer exciton diffusion length^9,10^, thus it is more attractive for fabrication of perovskite solar cells. Unfortunately, due to the specific characteristics of MAPbI_x_Cl_3−x_ perovskite, it is difficult to prepare smooth and dense perovskite films with fast and efficient method. Up to now, most of MAPbI_x_Cl_3−x_ perovskite layers were obtained by solution casting^11–13^. Totally, there are two types of solution casting methods: 1) one-step casting, the primary problem is that the film shrinkage would occur during the crystallization of perovskite due to the removal of solvent; 2) two-step casting, the main challenge is the volume expansion of PbI_2_ precursors because of the MAI intercalation. Thus, a few cracks or pinholes always appear in the perovskite films deposited by solution casting, leading to the deteriorated quality of the films and the resulting devices^14–18^. Vapor deposition technique is a promising alternative technique for preparing high-quality MAPbI_x_Cl_3−x_ perovskite films. Snaith *et al*. fabricated centimeter-scale MAPbI_x_Cl_3−x_ films with superior uniformity by a dual-source vapor deposition^19^. However, it is hard to control the precursor ratios precisely for obtaining high-quality perovskite films because of the complicated mechanism during the growth process. A layer-by-layer vapor-phase deposition approach was also developed to prepare high-quality MAPbI_x_Cl_3−x_ perovskite films to avoid the difficulty in controlling the precursor ratios^20^. The PSCs fabricated by this method showed an efficiency up to 14.29% while the film’s thickness can be precisely controlled by the deposition cycles. However, a long period was required in this method due to the limited reaction interfaces. Li *et al*. prepared MAPbI_x_Cl_3−x_ perovskite film using alternating precursor layer vacuum deposition and the device shows high performance and good stability. However, the relatively long period remains to be a major problems as described above^21^.

**Figure 1:**
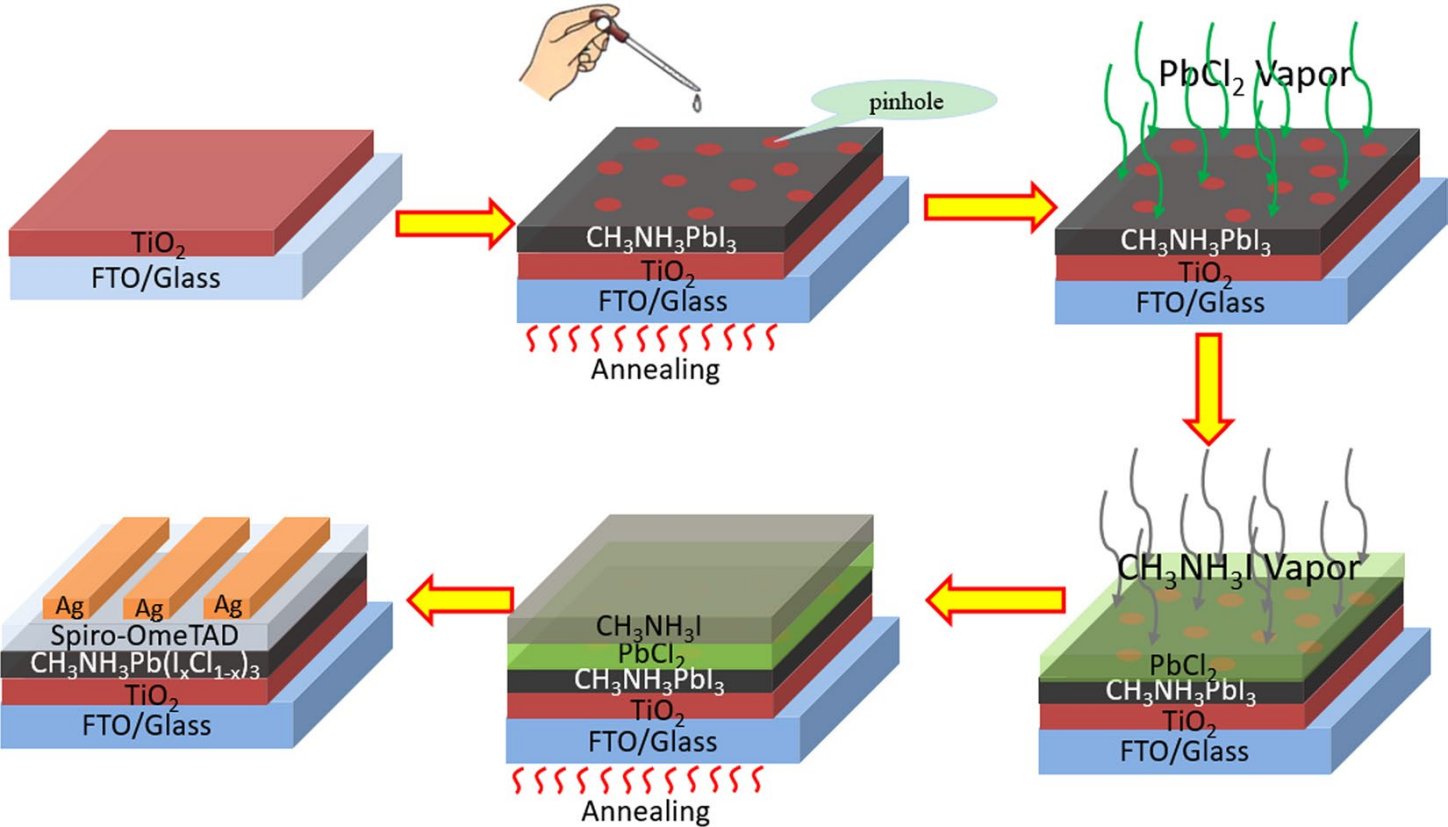
Schematic illustration on the formation of perovskite films through VSHM.

Herein, we developed a facile and effect method to overcome the shortcomings of the traditional methods as described above. In order to avoid the complicated process in traditional vapor deposition, we developed an optimized vacuum deposition solution hybrid method (VSHM) to fabricate perovskite thin films as showed in Fig. 1, in order to accelate the fabrication of perovskite films while remain a smooth surface and full coverage. The VSHM route mainly involves four steps: i) fabricating MAPbI_3_ perovskite film by solution casting on the TiO_2_/FTO glass (this process often results in films with pinhole formation and incomplete surface coverage); ii) forming the inorganic framework film by vacuum depositing PbCl_2_; iii) depositing the organic CH_3_NH_3_I by vacuum depositing on the PbCl_2_ films; iv) depositing hole transport material (HTM) and counter electrodes to form complete PSCs.

## Results and Discussions

**Figure 2:**
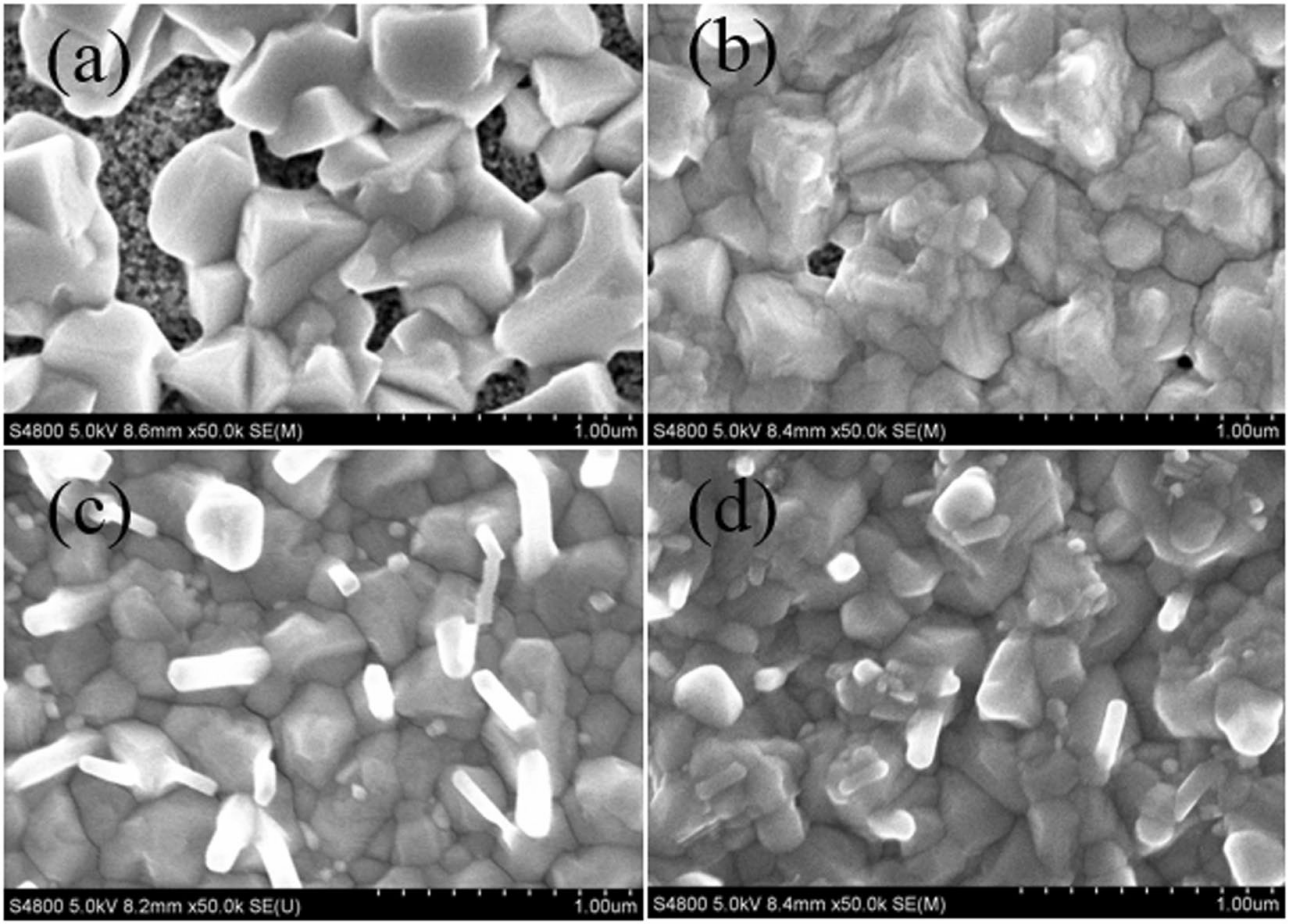
FESEM images of top view of MAPb(I, Cl)_3_ films with thickness of (**a**) 20 nm, (**b**) 40 nm, (**c**) 60 nm, and (**d**) 80 nm of the evaporating PbCl_2_.

It is critical to control the thickness of PbCl_2_ layers for a complete transformation of PbCl_2_ into uniform MAPb(I, Cl)_3_ perovskite layer by reacting with CH_3_NH_3_I. Obviously, thick PbCl_2_ layers would cause an incomplete conversion of the compact PbCl_2_ films into MAPb(I, Cl)_3_ perovskite. In other words, a PbCl_2_ layer would be residual, which deteriorates the film quality and hampers the device performance. Figure 2 shows the SEM images of the MAPb(I, Cl)_3_ films optimized using VSHM, by depositing PbCl_2_ layers with the thickness of 20 nm, 40 nm, 60 nm and 80 nm, respectively. As shown in Fig. 2a, the surface presented many large pinholes between grain boundaries (the deposition thickness is 20 nm) and the pinholes gradually decreased with increasing the PbCl_2_ thickness. In Fig. 2b, the coverage of the films has been improved obviously, only a few pinholes still remain in the films with the size of about dozens of nanometer. When the PbCl_2_ thickness increased to 60 nm and 80 nm, the pinholes disappear completely with the full coverage of perovskite films. It is note worthy that there are some dots with sizes of tens of nanometers on the surface of 60 nm and 80 nm thickness PbCl_2_ layers. The tiny dots might be reactive “nuclei” for the growth of grains, originating from the reaction between PbCl_2_ and MAI vapor. With the presence of the newly formed perovskite crystals on the top, along with the “nuclei” decorated around, we believe that the intercalation reaction takes place on the top of the PbI_2_ films in this stage^22^.

**Figure 3:**
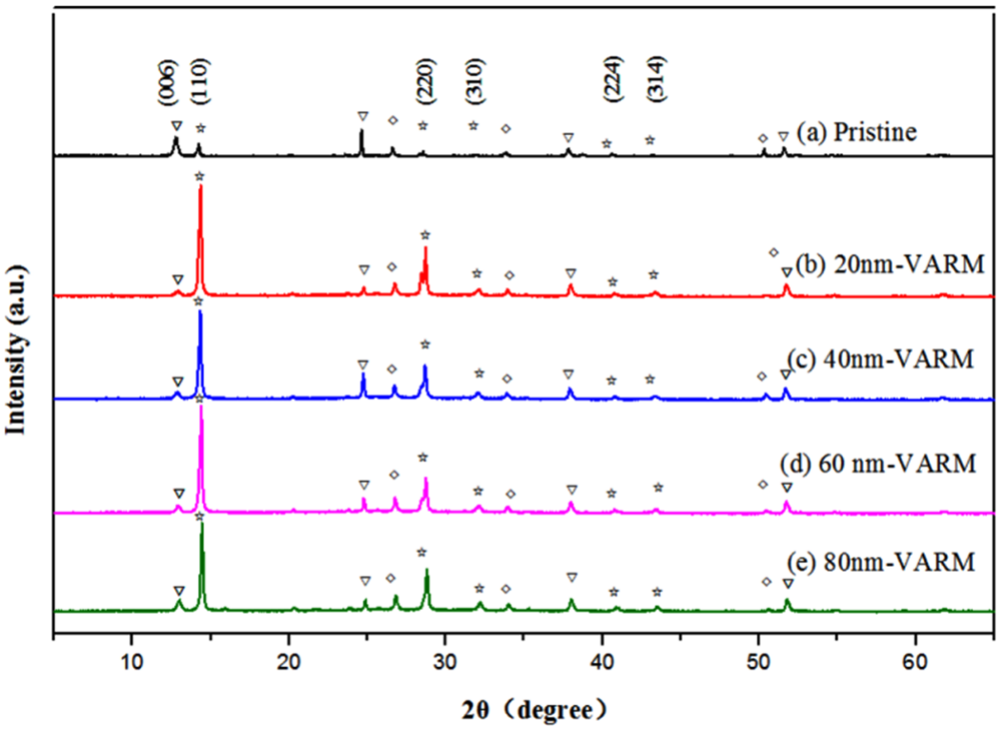
XRD patterns MAPb(I, Cl)_3_ films. Peaks associated with the perovskite, the PbI_2_, and the TiO_2_ are labelled (☆is for perovskite structure, ▽ is for PbI_2_, and ◇ is for TiO_2_.).

Figure 3a shows the corresponding XRD patterns of MAPbI_3_ films on FTO/c-TiO_2_ substrate prepared by the solution-based method. A set of peaks at 14.08°, 28.41°, 31.85°, 41.25° and 43.19° could be assigned to the planes (110), (220), (310), (224) and (314) of the MAPbI_3_, respectively. Additionally, the XRD patterns of VSHM perovskite films are showed in Fig. 3b–e. The relative intensities of the MAPb(I, Cl)_3_ peaks are increased compared to the pristine films after optimizing by VSHM, indicating the improved the crystallinity of perovskites. Compared with the solution method, the enhanced (110) and (220) peaks in Fig. 3 for the as-deposited MAPb(I, Cl)_3_ perovskite coating indicates an increased orientation of crystalline domains by our VSHM. In short, the uniform MAPb(I, Cl) perovskite films with oriented crystalline domains ensure a high charge mobility necessary for high-performance solar cells^23^.

**Figure 4:**
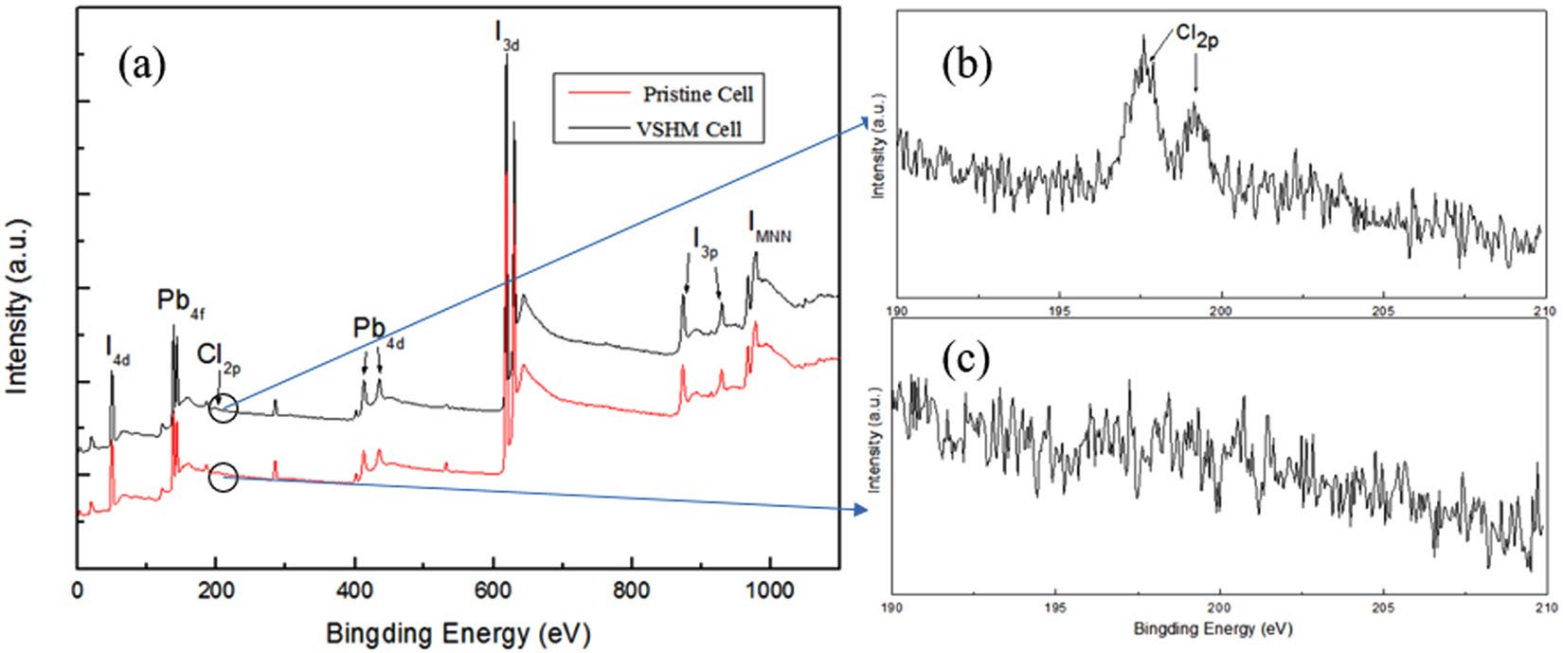
(**a**) XPS spectra of the pristine and VSHM based samples; the corresponding Cl_2p_ scans in pristine (**b**) and VSHM samples (**c**).

The Cl doping plays a key role in regulating the dynamics of exction/charge carrier transport in the MAPb(I, Cl)_3_ perovskite. Figure 4 shows a survey XPS spectrum for the MAPbI_3_ pristine perovskite and the MAPb(I, Cl)_3_ VSHM perovskite. As can be seen, the Cl element has been successful incorporated into the perovskite films via VSHM. In details, the Cl_2p_ scan for the VSHM-based perovskite and pristine perovskite were shown in Fig. 4b,c. Apparently, the peaks located at 197.64 and 199.14 eV could be found in the scanning spectra of Cl_2p_.

The J-V curves of the VSHM solar cells are shown in Figure S1. The corresponding photovoltaic parameters were summarized in Table S1, including the short-current density (J_sc_), the open-circuit voltage (V_oc_), the fill factor (FF) and the power conversion efficiency (PCE). There are 12 individual devices fabricating under different conditions. Compared to the pristine cells, the J_sc_ of 20 nm thick VSHM cells are improved evidently, from 17.46 to 19.88 mA cm^−2^. Result from the existence of cracks and pinholes in the pristine perovskite cells as shown in Fig. 2, which could be repaired by the PbCl_2_ vapor. When the thickness is increased from 20 to 40 nm, the J_sc_ and V_oc_ get increased simultaneously, owing to the optimized quality of the perovskite films from either the reduced defects or the improved surface coverage. Both of them are benefited for hindering electron recombination and increasing the PCE to 12.50%. When the PbCl_2_ thickness is increased to 60 nm, it can be observed from Figure S1 that the V_oc_ is increased a little bit, which is due to the 60nm-VSHM films have a full coverage than the above cells. The J_sc_ of the 60nm-VSHM cells are decreased obviously compared with 40nm-VSHM cells, leading to a PCE of 10.43%. This may be resulted from two reasons as following: Firstly, 60 nm thick PbCl_2_ films by vapor deposition are so dense that some PbCl_2_ are residue, which are harmful for electronic transport; secondly, when the thickness is up to 60 nm, there are lots of tiny dots on the surface of the perovskite films (Fig. 2c,d). It is speculated that these tiny dots are reactive PbCl_2_ nuclei which will increase the interface resistance between perovskite layer and HTM layer to hinder the electron transport. With the thickness of PbCl_2_ films increasing to 80 nm, the PCE of the devices further decreases to 9.13%. This illustrated that the films are too thick (>60 nm) for preparing perovskite absorbed layer, of which the reasons are similar to the 60nm-VSHM cells as described above. Although the PCE values of the cells optimized by VSHM are obviously improved than the pristine devices, it also can be further optimized by control the PbCl_2_ films by vapor deposition more accurately. It’s because that there are less number of pinholes in 20 and 40 nm-VSHM perovskite layers than those in the 60 nm-VSHM perovskites, owing to an incomplete transformation of PbCl_2_. According to the above, we speculate that the optimal PbCl_2_ thickness should be between 40 nm and 60 nm. Next, the optimal 50 nm-VSHM perovskite (called “VSHM perovskite” for abbreviation in latter section) films and subsequently PV devices will be discussed in detail.

**Figure 5:**
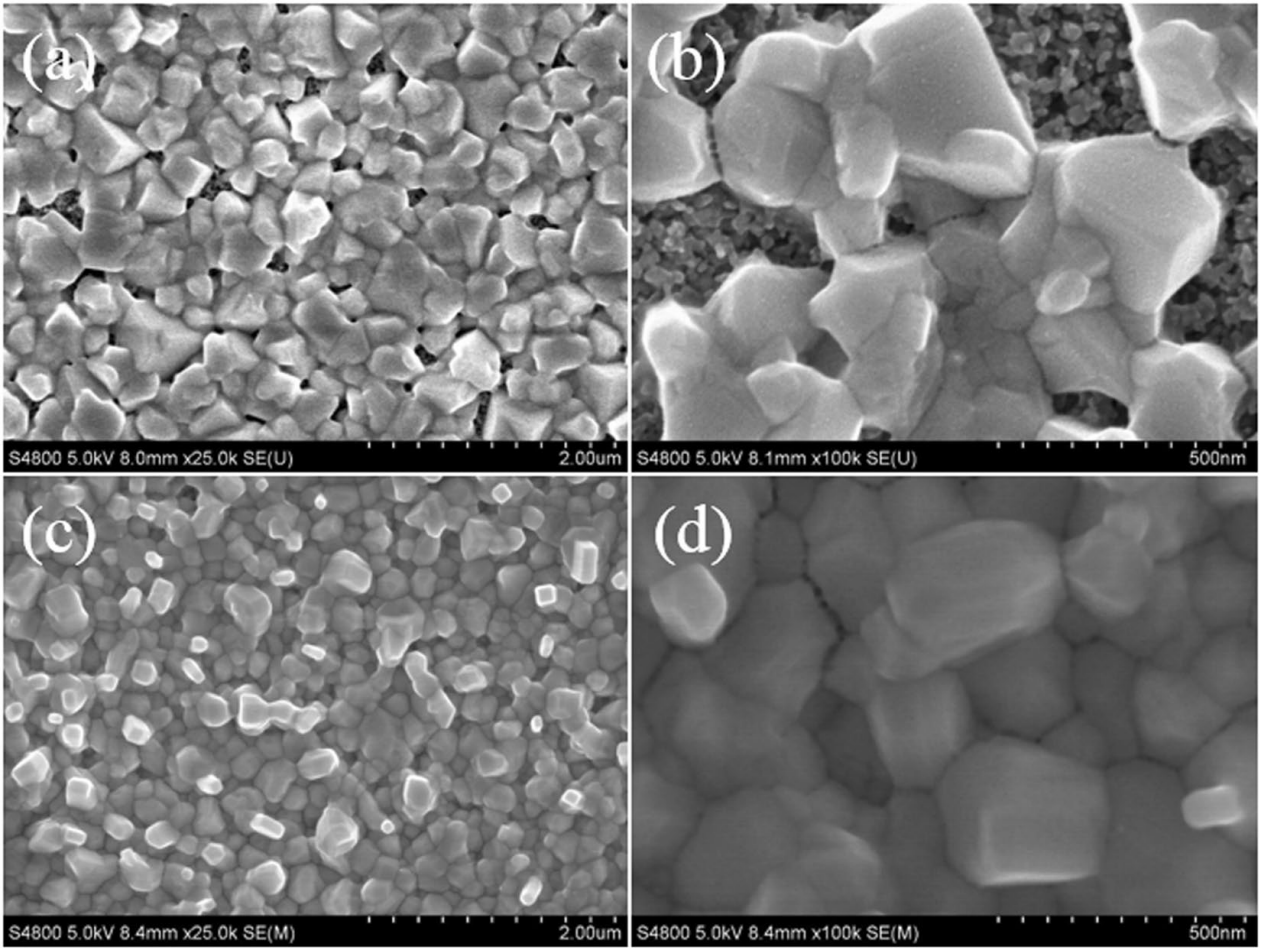
Top view FESEM images of pristine perovskite films (**a**,**b**) and VSHM perovskite (**c**,**d**).

Figure 5a,b shows the SEM image of pristine perovskite films with different magnification. It can be seen that lots of pinholes appeared between grains. In particular with Fig. 5b, we can see that the perovskite films exhibit poor coverage so that TiO_2_ porous layers are exposed. Figure 5c,d shows the SEM of perovskite films prepared by VSHM under the optimized conditions (50 nm). The pinholes nearly disappeared in perovskite films in Fig. 5c. From Fig. 5d, we can see the almost perfect perovskite which presents uniform morphology and density with a full coverage. The size of the perovskite grains are about 200–300 nm. In a word, the quality of perovskite films can be obviously improved by VSHM, due to the larger grains and the more full coverage.

**Figure 6:**
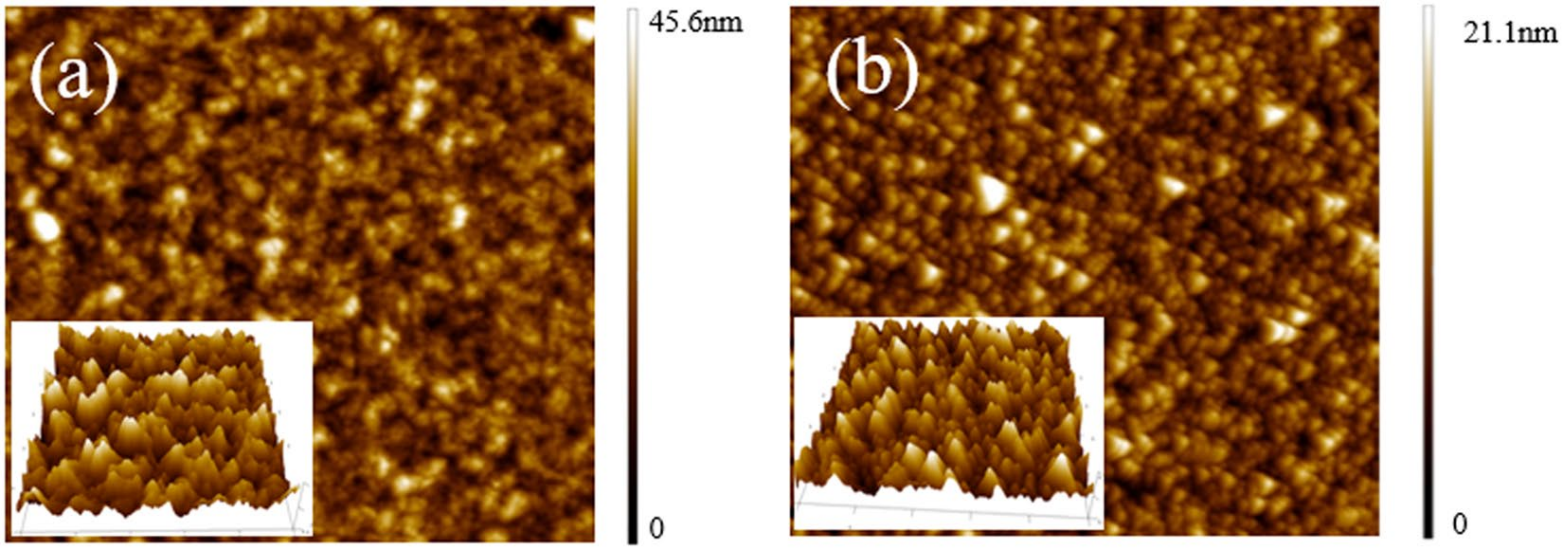
AFM images of pristine (**a**) and VSHM perovskite film (**b**).

Figure 6 shows the AFM images of pristine and VSHM perovskite films. The surface of pristine perovskite film is rough with an average roughness of about 45.6 nm. Compared to pristine perovskite films, the VSHM perovskite films are smoother with a smaller surface roughness of about 21.1 nm. This may be due to a smooth PbCl_2_ film as well as the repairing of the pinholes inside the perovskite films. Small surface roughness is benefited for depositing HTM layers and reducing the interface resistance to accelerate the charge transport^24^.

**Figure 7:**
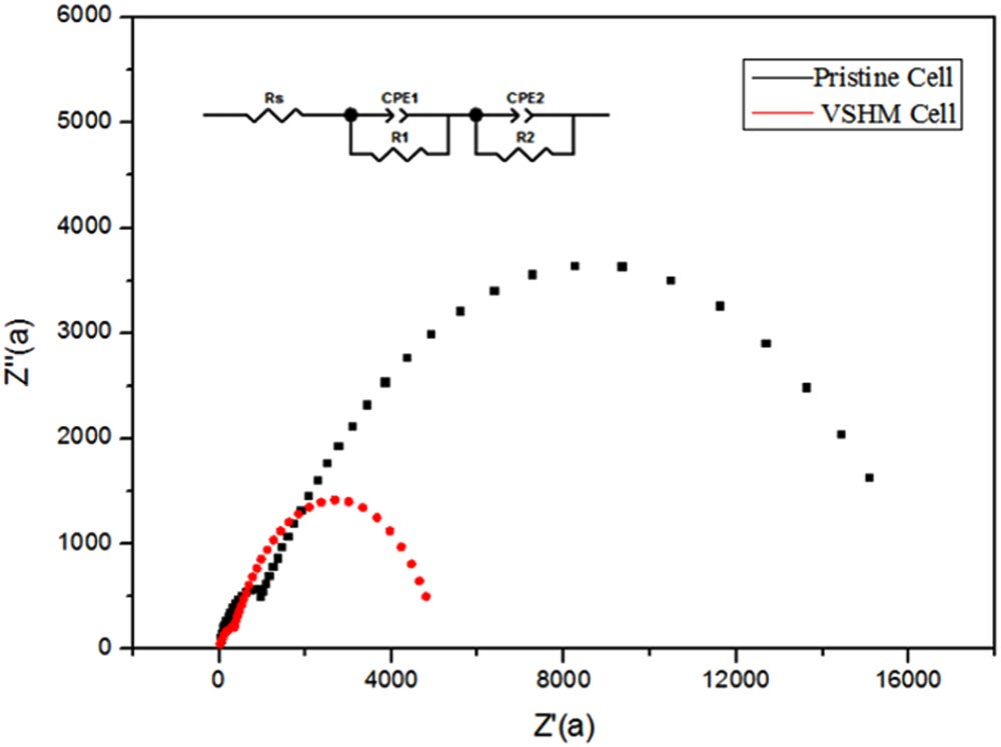
EIS spectra of pristine and VSHM PSCs.

In order to investigate the electrical properties of the interfaces distinction between pristine and VSHM cells, the EIS measurement was performed in dark. As shown in Fig. 7, the Nyquist plot is usually composed of two irregular semicircles, including the small one at high frequency and the large one at low frequency, and the equivalent circuit is inserted in Fig. 7 ^25–27^. The high frequency (corresponding to low Z’) intercept on the real axis (Z’ axis) represents the series resistance R_s_, which is mainly associated to the resistance of FTO substrate^28^. Specifically, the semicircle in the high-frequency range results from the charge transfer resistance (R_1_) occurring at the perovskite/HTM interfaces, while the semicircle in the low-frequency range results from the charge recombination resistance (R_2_) at the TiO_2_/perovskite interface. From Fig. 7, the R_s_ and R_1_ of VSHM cells are nearly the same to that of the pristine cells, suggesting the series resistance is not increased by VSHM. The main difference lies in the R_2_ of perovskite devices. It could be observed that VSHM perovskite devices present a much higher resistance (R_2_) than the pristine one. The increased resistance implies a lower charge recombination at TiO_2_/perovskite interface. Because of the low carrier recombination rate at TiO_2_/perovskite interface, it is reasonable that VSHM cells show both higher J_sc_ and V_oc_ than those of the pristine one.

**Figure 8:**
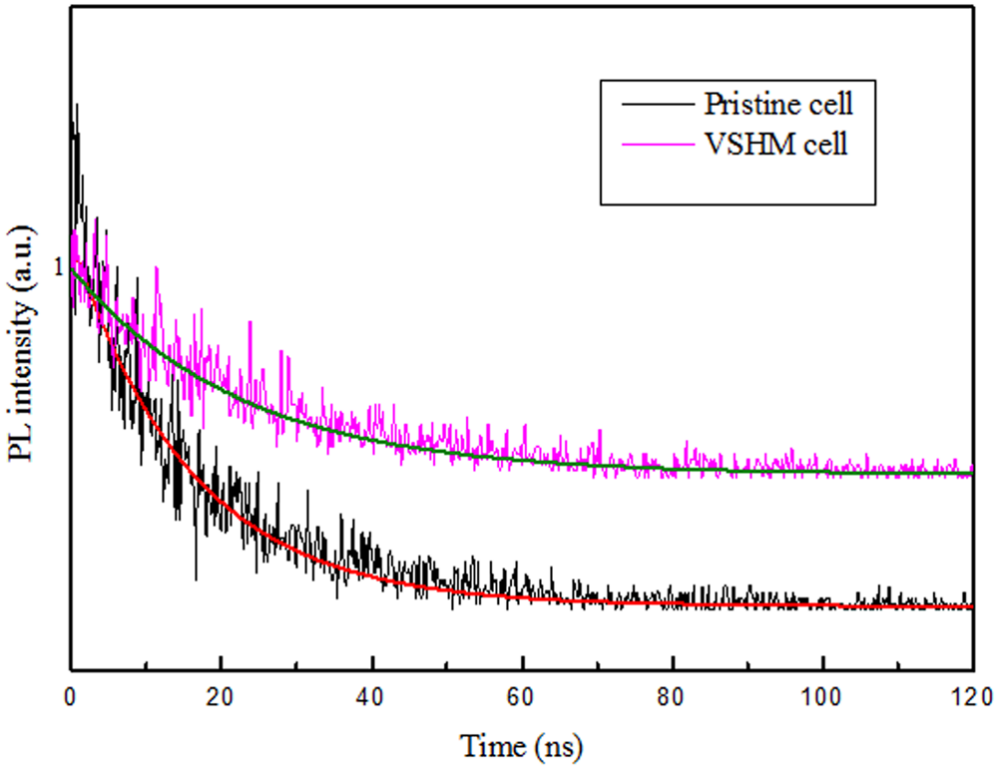
Transient PL spectra of the pristine and VSHM cells.

As can be seen in Fig. 8, transient PL spectra are collected to further investigate the charge transport properties of the perovskite films derived from different deposition methods, including solution method (pristine cell) and VSHM method. Herein, the charge extraction capability of the MAPb(I, Cl)_3_ perovskite with VSHM is obviously superior to that of the pristine MAPbI_3_ film. The corresponding simulation curves of the cells fitted with a double exponential function are also shown in Fig. 8. From the fitted curves, the charge lifetime of the MAPb(I, Cl)_3_ perovskite fabricated via VSHM is estimated to be 20.7 ns, which is remarkably longer than the pristine cell (14.1 ns). Base on the previous reports, both the exctions diffusion length and the charge mobility have been reported to be significantly improved by the Cl doping^9^. On the other hand, the optimized surface morphology of VSHM films with full coverage is conducive to prevent charge recombination between perovskite films and charge transport layers.

**Figure 9:**
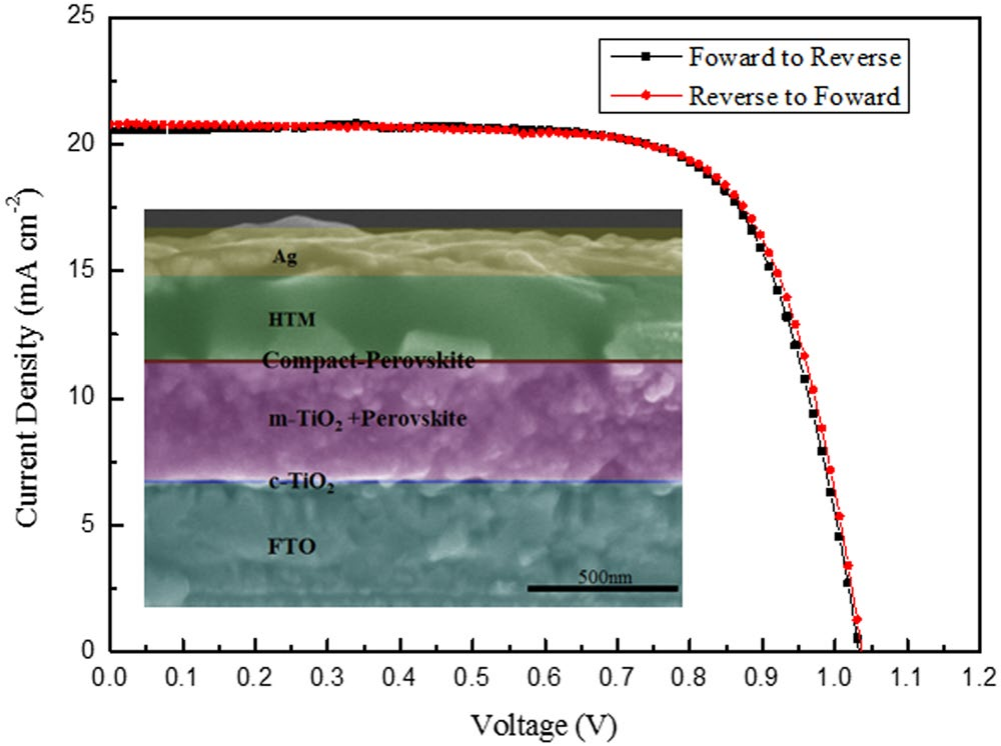
J-V curves of the optimized VSHM perovskite cell.

The J-V curves of the PSCs with an optimized parameters are shown in Fig. 9, where a cross-sectional SEM image of the perovskite cell is inserted. The corresponding photovoltaic parameters were summarized in Table S2, including the short-current density (J_sc_), the open-circuit voltage (V_oc_), the fill factor (FF) and the power conversion efficiency (PCE). The PCE of 50-nm VSHM cells are improved evidently to 15.57%, which is the highest PCE value in all of the VSHM cells. The reasons can be speculated as following: when the PbCl_2_ thickness is 50 nm, the perovskite layer is continuous without any pinholes, suggesting that 50 nm PbCl_2_ is thick enough to form a full coverage. Note that, unlike 60 nm or 80 nm VSHM perovskite, no “nuclei” appears in 50 nm VSHM perovskite, this will be helpful for the charge transportation.

PSCs are known to show an anomalous hysteresis in the J-V curves, making it difficult to characterize the device performance accurately. To ensure the accuracy of our characterization, the devices are investigated using different scanning directions as are shown in Fig. 9, in which no hysteresis is observed in the J-V curves. It suggests that the PCE measured from the J-V curves of our MAPb(I, Cl)_3_ perovskite solar cells are reliable. Such a hysteresis-free characteristic could be attributed to a low surface defect density of the perovskite films.

**Figure 10:**
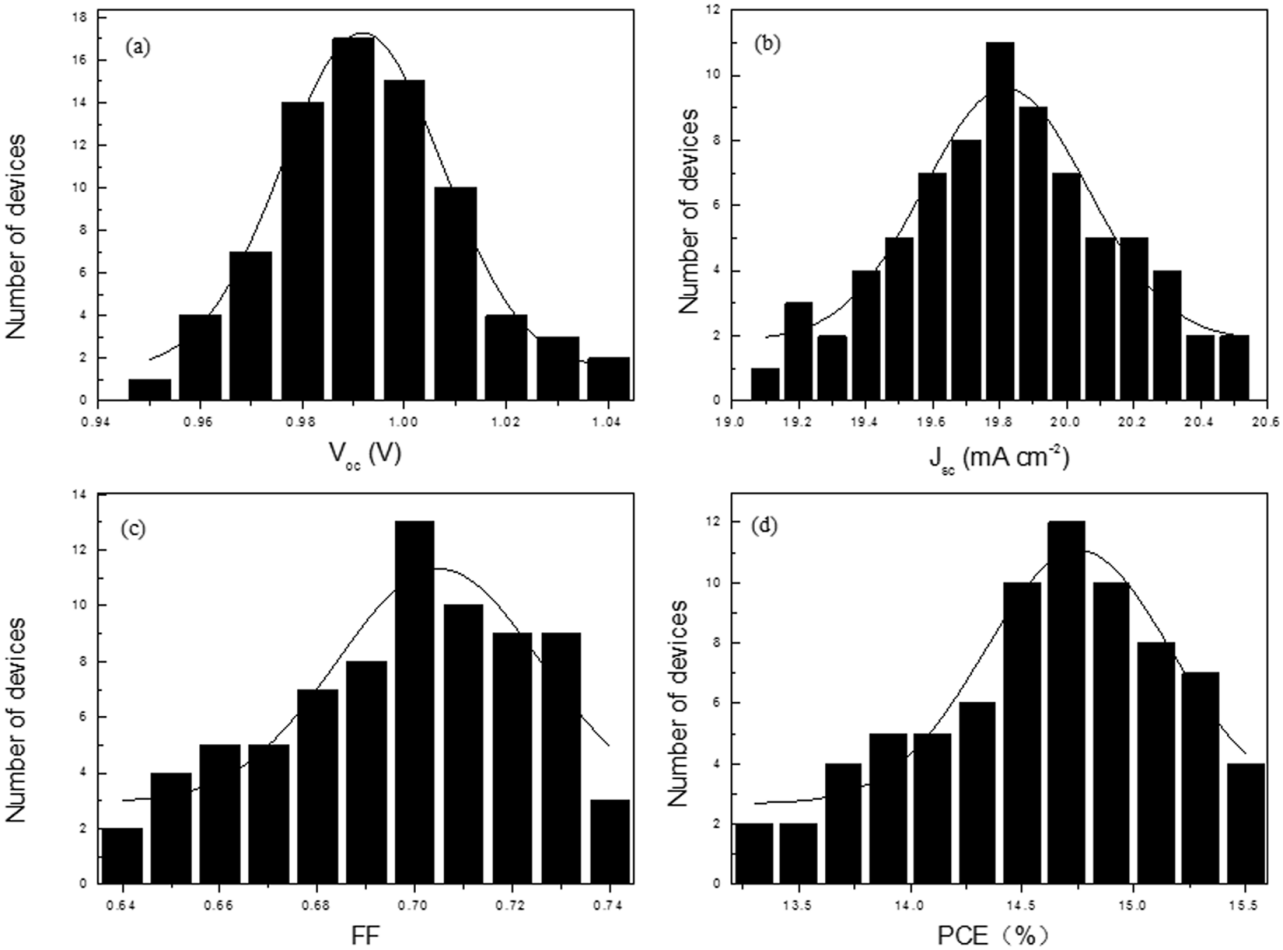
Histograms of device performance for VSHM perovskite cell. (**a**) V_oc_, (**b**) J_sc_, (**c**) FF and (**d**) PCE measured for 75 individual devices.

**Figure 11:**
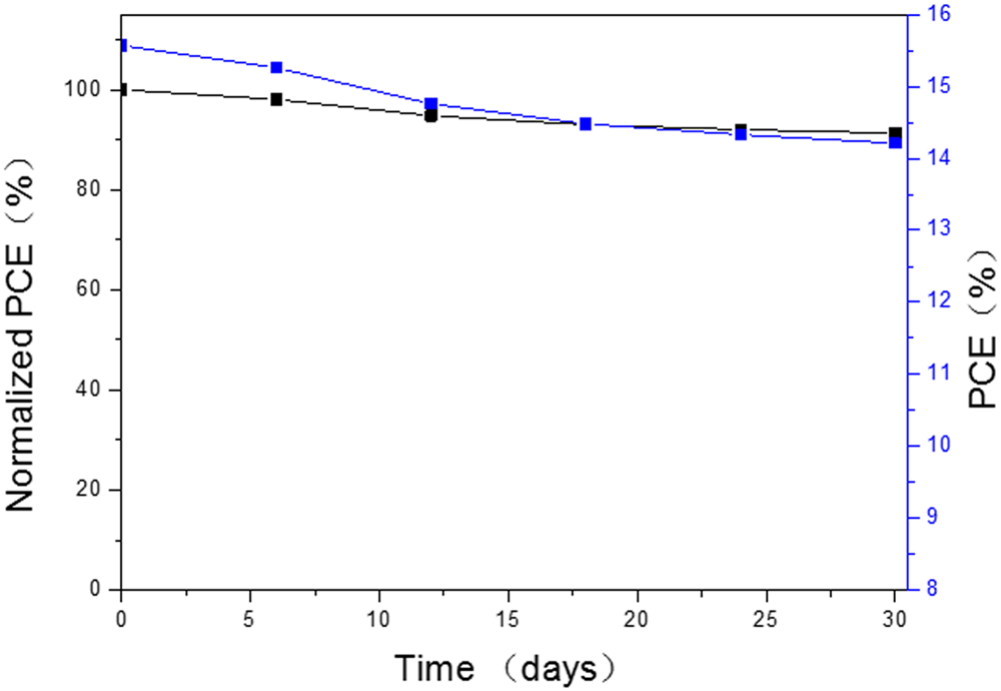
PCE stability of the VSHM cells without encapsulation stored under ambient conditions.

Moreover, our results indicate that high performance perovskite solar cells can be repeatedly fabricated using the VSHM route. Histograms of the V_oc_, J_sc_, FF, and PCE of 75 individual devices using the optimized layer thickness (50 nm) are given in Fig. 10, which show good reproducibility with a typical V_oc_ value of 0.98 V, 19.80 mA cm^−2^ for J_sc_, 0.70 for FF, and over 14% for PCE for more than 82% of the devices. In addition, the device fabricated by VSHM shows superior stability. Using a bare device without any encapsulation, we measured the J–V curves on a daily basis for 30 days under ambient conditions (humidity: ≤80%, temperature: ≤35 °C). Very good stability was demonstrated. Specifically, the PCE value remained over 90% of its initial efficiency after 30 days, as shown in Fig. 11. The parameters are listed in Table S3. We attribute such a remarkable stability to the full surface coverage of the dense perovskite films which facilitates preventing the moisture penetration.

In this study, we have developed VSHM, a facile and effective technique to fabricate perovskite films and the corresponding PV devices. The perovskite film derived from this approach exhibits smooth surface and full surface coverage. The obtained VSHM cells exhibited an average efficiency of the planar device is 13.42% with a standard deviation of ±2.15% and best efficiency as high as 15.57% and superior stability with the PCE value remained over 90% of its initial efficiency after 30 days. In a word, the VSHM has been demonstrated to be a versatile and controllable approach to the pursuit of high-quality perovskite films and the resulting high-performance, repeatable and stable PV devices.

## Method

### Synthesis of CH_3_NH_3_I

Methylammonium iodide (MAI) was synthesized by reacting methylamine (33 wt% in absolute ethanol, J&K) and hydroiodic acid (57 wt% in water with 1.5% hypophosphorous acid, J&K) at a 2: 1 molar ratio in ethanol in a 250 mL round bottom flask under nitrogen at 0 °C for 2 hours with stirring. After reaction, the white precipitate of MAI was recovered by rotary evaporation at 60 °C for 1 hour and then dissolved in ethanol. The pure MAI was recrystallized from ether and dried at 60 °C in a vacuum oven for 48 hours.

### Device fabrication

Fluorine doped tin oxide glass substrates (FTO, Pilkington, TEC 15) were patterned by etching with Zn powder and 4 M HCl diluted in deionized water. The etched FTO substrates were subsequently cleaned by ultrasonication in a detergent solution, deionized water, acetone and isopropanol respectively, then rinsed with deionized water, ethanol and dried with clean dry air^29–32^. A dense blocking layer of TiO_2_ was deposited onto the substrate by spin-coating a TiO_2_ colloid solution at 3000 rpm for 30 seconds and then annealed at 450 °C for 1 hour. For the mesoporous TiO_2_ film, TiO_2_ paste (Dyesol, 18 NRT) was used in absolute ethanol in the ratio 1: 3.5 by weight, which was spin coated on the FTO/c-TiO_2_ substrates at 5000 rpm. for 30 seconds, followed by drying at 130 °C for 10 minutes sintering at 500 °C in air for 15 minutes. Two-step solution chemistry method was used to fabricate the underlayer CH_3_NH_3_PbI_3_ inside a glove box (Mikrouna, Universal). In briefly, PbI_2_ were dissolved in DMF (500 mg/mL) at 70 °C and spin-coated on the FTO/TiO_2_ substrates at 5000 rpm for 5 seconds then dried at 70 °C for 30 minutes. Upon cooling, CH_3_NH_3_I was dissolved in iso-propanol solution (10 mg/mL) at 70 °C dropped on FTO/TiO_2_/ PbI_2_ substrates for 120 seconds and spin-coated at 5000 rpm for 30 seconds. They were dried by spin-coating at 3000 rpm for 20 seconds and by heating at 70 °C for 30 minutes. For the VSHM samples, they were transferred into the evaporator which was inside the above glove box for PbCl_2_ and CH_3_NH_3_I evaporation. The thickness of the PbCl_2_ and CH_3_NH_3_I films were adjusted by the temperature and time of the evaporation. Full details of the evaporation are provided in the supporting information. Then, they were dried by heating at 70 °C for 30 minutes. The FTO/TiO_2_/Perovskite substrates were deposited by spin-coating a HTM layer solution at 3000 rpm for 30 seconds, where a spiro-OMeTAD (BoRun)/chlorobenzene (73.2 mg mL^−1^) solution was employed with addition of 17.5 µL Libis (trifluoromethanesulfonyl) imide (Li-TFSI, aladdin)/acetonitrile (520 mg mL^−1^) and 28.8 µL tert-butylpyridine (TBP, Sigma). Finally, the counter electrode was deposited by thermal evaporation of silver under a pressure of 8 × 10^−5^ Torr. The active area of the cell was measured to be 0.1 cm^2^ which was masked by a black tape during the testing of the cells.

### Device characterization

X-ray diffraction spectra (XRD) were obtained from the perovskite samples deposited on the FTO/TiO_2_ substrates with a Bruker D8. Data were collected using a Bruker D8 Advance diffractometer fitted with a Cu-K_α_ source operated at 40 kV and 30 mA, a 0.6 mm divergence slit, a 8 mm anti-scatter silt, a Ni filer for Cu-K_β_ radiation, a 2.82° detector opening, a Primary soller slit secondary soller slit and a Lynxeye silicon strip detector. X-ray photoelectron spectroscopy (XPS) were also obtained from the perovskite samples deposited on the FTO/TiO_2_ substrates with ThermoEscalab 250 Xi (USA). The XPS source gun type with Al-K_α_ source and a 0.9 mm spot size. The surface morphology of samples were characterized by field emission scanning electron microscope (FESEM, Hitachi S-4800, Japan). Atomic force microscope (AFM) was performed using Bruker dimension icon scanning probe microscope (SPM) in “tapping” mode. UV–visible absorption spectra (UV-Vis-NIR, Shimadzu 3150, Japan) were used to study the optical properties perovskite. The photocurrent density–voltage (J–V) measurements were carried out under illumination of 100 mW cm^−2^ using a Keithley model 2420 digital source meter. A solar simulator was used to simulate sunlight by a 500 W xenon lamp light source fitted with an AM1.5 G filter (Newport Oriel Sol3A, 94023). The illumination intensity of 100 mW cm^−2^ was calibrated with a standard monocrystalline silicon solar cell which passed the American Society for Testing and Materials (ASTM) calibration. The devices were measured between 0 V and 1.2 V under reverse or forward scan with the step voltage of 10 mV and the delay time 40 ms, respectively. The J–V curves for all devices were measured by masking the active area with a metal mask (0.1 cm^2^). Electrochemical impedance spectroscopy (EIS) was conducted by using electrochemical test station (Zahner, IM6ex).

## Electronic supplementary material

Supporting Information
