# Supplementary material for: Surface engineering of perovskite films for efficient solar cells
Source: Sci Rep. 2017 Nov 3;7:14478. doi: 10.1038/s41598-017-14920-w (PMC5670249; doi:10.1038/s41598-017-14920-w)
Supplement: Supplementary file 1 — Supporting Information [file 41598_2017_14920_MOESM1_ESM.pdf]

Supporting Information

Surface engineering of perovskite films for efficient solar cells

Jin-Feng Wang, Lei Zhu*, Ben-Guang Zhao, Yu-Long Zhao, Jian Song, Xiu-Quan Gu, Ying-Huai Qiang*

School of Materials Science and Engineering, China University of Mining and Technology, Xuzhou 221116, China

***Corresponding author's E-mail:** lzhu@cumt.edu.cn yhqing@cumt.edu.cn

Experiment

Evaporation of $PbCl_2$ and CH_3NH_3I

The $PbCl_2$ were deposited through evaporation onto the perovskite-TiO_2 coated FTO substrates under high vacuum. Vapour-deposition source was monitored using a quartz crystal monitor positioned a short distance from the source. The source to monitor distance is different to the source to substrate distance, and so a tooling factor was tested before evaporation. The device substrates were placed in a substrate holder above the sources with the perovskite-TiO_2 coated FTO side facing down towards the sources. Once the pressure in the chamber was pumped down to below 8×10^{-4} mbar, the source was heated to about 325°C for approximately 10 min to remove volatile impurities before depositing the materials onto the substrate. The substrate holder was rotated to ensure uniform coating throughout deposition. For the CH_3NH_3I, it was fabricated on glass/FTO/TiO_2/$PbCl_2$ substrate which the source was heated to about at 110 °C through evaporation. The thickness of the $PbCl_2$ and CH_3NH_3I films were adjusted by the temperature and time of the evaporation.

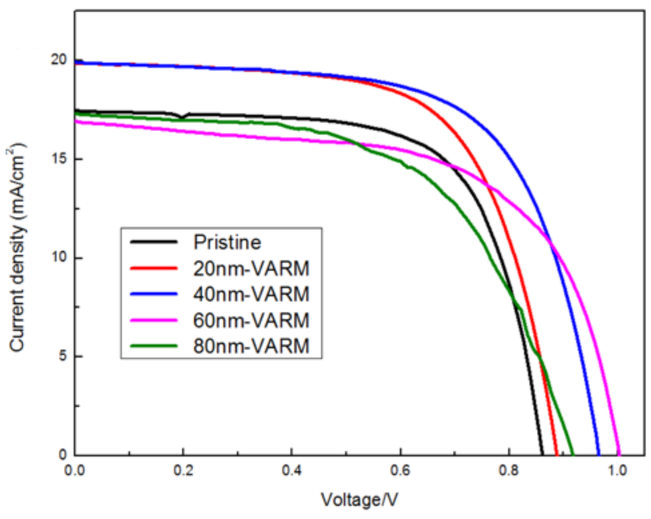

Figure S1. The J-V curves of perovskite solar cell with different thickness of $PbCl_2$.

Table S1 The J-V curves of the perovskite solar cells before (a) and after (b) optimized by SAVM

samples	J_{sc} (mA /cm^{-2})	V_{oc} (V)	FF(%)	PCE(%)
Pristine	17.46	0.86	67.92	10.20
20nm - VARM	19.88	0.88	65.79	11.51
40nm-VARM	19.92	0.97	64.74	12.51
60nm-VARM	16.94	1.00	61.57	10.43
80nm-VARM	17.32	0.92	57.30	9.13

Table S2 The J-V curves of the perovskite solar cells before (a) and after (b) optimized by SAVM

Samples	J_{sc} (mA /cm^{-2})	V_{oc} (V)	FF(%)	PCE (%)
Foward to Reverse	20.18	1.02	70.21	15.24
Reverse to Foward	20.50	1.03	73.34	15.57

Table S3 The parameters of perovskite solar cell without encapsulated stored in ambient.

Days	J_{sc} (mA /cm^{-2})	V_{oc} (V)	FF(%)	PCE(%)
0	20.50	1.03	73.34	15.57
6	20.43	1.02	73.23	15.26
12	19.97	1.02	72.46	14.76
18	19.68	1.01	72.84	14.48
24	19.55	1.00	73.30	14.33
30	19.43	1.01	72.41	14.21
